# Euler–Lagrange Simulations of Microstructured Bubble Columns Using a Novel Cutting Model

**DOI:** 10.1021/acs.iecr.3c02352

**Published:** 2023-09-12

**Authors:** Rahul Subburaj, Yali Tang, Niels G. Deen

**Affiliations:** † Power and Flow Group, Department of Mechanical Engineering, Eindhoven University of Technology, P.O. Box 513, 5600 MB Eindhoven, The Netherlands; ‡ Eindhoven Institute for Renewable Energy Systems (EIRES), Eindhoven University of Technology, P.O. Box 513, 5600 MB Eindhoven, The Netherlands

## Abstract

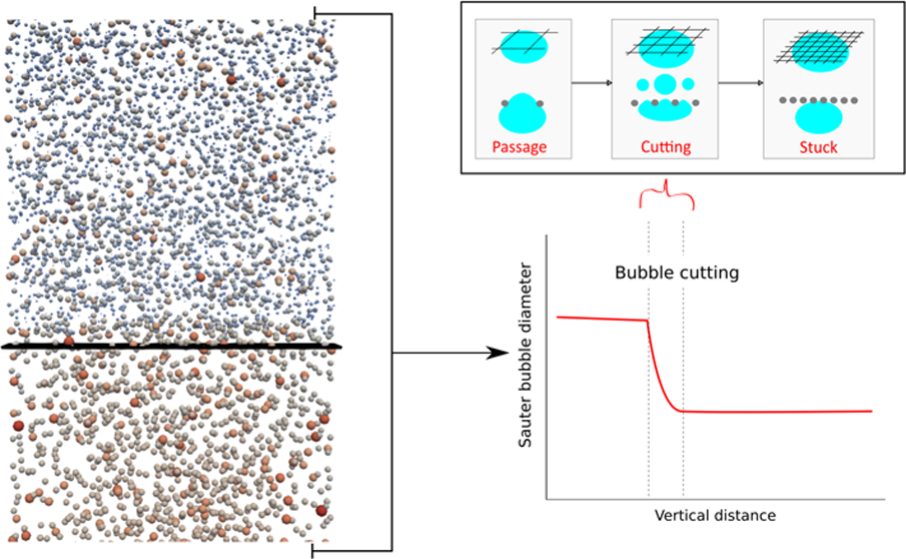

In the concept of a microstructured bubble column reactor, meshes coated with catalyst can cut the bubbles, which in turn results in high interfacial area and enhanced interface hydrodynamics. In previous work, we developed a closure model for the fate of bubbles interacting with a wire mesh based on the outcomes of energy balance analysis. In this paper, the model is validated using Euler–Lagrange simulations against two experimental cases of microstructured bubble columns. Before validation of the model, the definition of the deceleration thickness, as used in the calculation of the virtual mass term, is refined to introduce the effects of liquid viscosity and wire thickness. Proceeding with the validation, the inclusion of our cutting closure model results in an excellent match of the bubble size reduction by the wire mesh with the experimental data. Consequently, the simulations produce a more accurate prediction of the reactor performance for the gaseous reaction in view of the pH and gas holdup profiles. The effect of liquid viscosity on the bubble size reduction by the wire mesh is replicated accurately as well. Noticeably, the significance of bubble coalescence and breakup in bubble dynamics overperforms the role of bubble cutting at high superficial gas velocities; thus, further improvement is needed there. Finally, based on the validated cutting model, we share some perspectives on the design of wire meshes to increase the bubble interfacial area.

## Introduction

The bubble column reactor (BCR) is a commonly used reactor in refineries and chemical and pharmaceutical industries due to its simplicity in design and lack of moving parts. Reactants are often injected as bubbles into a slurry of catalysts and products. In such a reactor, multiscale phenomena such as fluid flow, mass transfer, and chemical reactions occur interdependently. Due to this, scaling up of BCRs is challenging as, e.g., a higher superficial gas velocity leads to stronger bubble coalescence resulting in a heterogeneous size distribution. Large bubbles can hinder the reaction yield due to the lower surface-to-volume ratio. Thus, strategies are needed to reduce bubble size and prevent coalescence. Several modifications such as sieve trays, structural packing, and vertical packing^1^ have been introduced to reduce gas/liquid back-mixing, thus achieving a uniform distribution of relatively small bubble sizes. Without any internal structure changes, it was demonstrated that the use of low-frequency vibrations^2,3^ and the addition of small particles^4,5^ can also influence the bubble size distribution and improve the rate of gas–liquid mass transfer. Many studies^6−10^ were performed on the so-called microstructured bubble column (MSBC), i.e., a bubble column with trays of fibrous catalyst material. These sieve trays not only provide catalysts for the reaction but also function to cut or break large bubbles. A general consensus has been reached in the literature with respect to this positive effect on the bubble size distribution. Some interesting observations were reported by Yang et al.^11^ who analyzed the interaction between rising bubbles and sieve trays or wire meshes. An increase in the drag force and breakup of the rising bubble was found, which strongly depends on the sieve pore size. Bubble breakup occurred only when the sieve pore size was larger than the Sauter mean diameter; otherwise, the bubbles just slowed down (due to increased drag). For smaller pore sizes, cutting is not straightforward, as the bubble gets stuck behind the mesh. Chen et al.^12^ observed a similar bubble size reduction but a reduced cutting for liquids of higher viscosity. Furthermore, Sujatha^13^ performed several experiments on an MSBC and found an increase in mass transfer coefficient as a result of bubble cutting/breakup. Therefore, it can be concluded that knowledge of the interaction between wire meshes and gas bubbles is a prerequisite to determining its effect on the conversion/yield of the MSBC, which however is extremely complex and yet unpredictable. Baltussen^14^ used direct numerical simulations (DNS) to study the effect of bubble Eötvös number (*Eo*) and wire mesh grid spacing on the outcome of bubble–wire interaction, i.e., bubble passage/cutting. A competition between the surface tension force (increased due to the effects of the wire mesh) and buoyancy was observed. In dimensionless terms, this could be summarized as larger bubbles with *Eo* > 4 being able to pass through, while smaller bubbles (*Eo* < 4) are able to pass only when the mesh spacing is larger than 0.625 times the bubble diameter. A liquid film between the bubble and wire was observed by Wang et al.^15^ upon performing DNS simulations of two bubbles of different volumes impacting a cylindrical wire. As a result, it can be maintained that bubble–wire interaction is independent of surface wettability effects, thus simplifying our study.

Computational fluid dynamics (CFD) is a powerful tool to further analyze and improve BCRs due to its simplicity and modular approach. Recently, Muniz and Sommerfeld^16^ presented an Euler–Lagrange numerical computational study, proposing different formulations for the different involved forces, i.e., drag, lift, and added mass, based on the instantaneous bubble eccentricity and compared the effect of the different force formulations. Taborda and Sommerfeld^17^ extended this model for reactive bubbly flow simulations, specifically for the two cases of CO_2_ rising in pure water and in a NaOH solution. A later study^18^ improved upon the lift modeling and dynamic Sherwood number to produce more accurate results. Hlawitschka^19^ enumerates the several multiscale phenomena in a bubble column reactor such as hydrodynamics, mass transfer, and reactions and provides a reliable modeling framework for simulations. Another study by Hlawitschka et al.^20^ implements an accurate representation of bubble motion, using an oscillation model, to implement the effects of liquid properties such as liquid viscosity.

For large-scale MSBC simulations, closure models are needed along with a general CFD framework to describe the bubble–wire interaction, accounting for the effective bubble cutting/breakup. The current work is interested in establishing a realistic model to describe the bubble cutting (interaction of bubble with wire mesh) behavior in MSBC simulations. Thus, we implement a simpler modeling of the three components (hydrodynamics, mass transfer, and reactions) without including the effects of bubble eccentricity and oscillations, similar to Huang et al.^21^ Jain et al.^22^ applied a simple geometrical cutting model to the Euler–Lagrangian simulations, in which the bubble is sectioned into several daughter bubbles depending on how much of the mother bubble is exposed to the individual mesh openings. This model works fairly well when the simulations are compared to the experimental results; however, it lacks a physical basis. In our previous work,^23^ an improved cutting model based on energy analysis was developed and validated with DNS results of single bubble–wire interaction by Baltussen.^14^ In this paper, we further derive one of the model parameters, the bubble deceleration thickness using lubrication theory. It is found that this thickness is dependent on the wire thickness and liquid viscosity. Subsequently, we test this cutting model for both reactive and nonreactive bubbly flow simulations using the Euler–Lagrangian framework. Two case studies are performed: chemisorption of NaOH with validation experiments by Sujatha^13^ and experiments with different liquid viscosities by Chen et al.^12^ Lastly, an optimization study is performed on the general case of bubble cutting to maximize the number of (cut) daughter bubbles.

## Numerical Framework

An open-sourced OpenFOAM-based solver is employed for this work, which combines the discrete particle method (DPM) and the volume of fluid (VOF) method. Note that VOF is used for capturing only the free surface in our simulations. As the numerical framework, including the turbulence model and subforce models, has been described and justified in our previous publication,^24^ we only provide a simplified description of the framework used in this work. The additional models, including coalescence and breakup, bubble cutting, and species and mass transfer, are further introduced.

### CFD-DPM Coupled with VOF

The volume-averaged Navier–Stokes equations are solved for the continuous phase
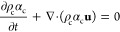
1
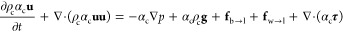
2where α_c_ is the volume fraction of the continuous phase, ρ_c_ is the fluid density, **u** is the fluid velocity, *p* is the pressure, **f**_b→l_ is the force density terms of the bubbles on the liquid, **f**_w→l_ is the force density term of the wire on the liquid, and **τ** is the stress tensor. Definitions of the different terms in the equations can be listed as

1. The continuous phase volume fraction α_c_ (for ’cell i’) is defined as, α_c_ = 1 – α_d_ – α_w_, where α_d_ is the volume fraction of discrete phase and is given by
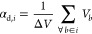
3with the cell volume Δ*V* = *ΔxΔy*Δ*z* and volume of bubble, *V*_b_ = *π d*_b_^3^/6, with *d*_b_ as the bubble diameter. Similarly, α_w_ is the volume fraction of the wire mesh.
2. The stress tensor (**τ**) constitutes of viscous and turbulent stress terms, **τ** = **τ**_l_ + **τ**_*t*_ = (ν + ν_sgs_) **D**, where **D** is the deformation tensor. Smagorinsky SGS model^25^ calculates the subgrid-scale kinematic viscosity as,
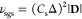
4where Δ = (Δ*V*)^1/3^ is the filter size. Zhang et al.^26^ suggested an appropriate *C*_s_ value of 0.1 to accurately predict the averaged *y*-velocity and turbulent stresses.
3. Force density terms (**f**_b→l_ & **f**_w→l_) in “cell *i*” are obtained by filtering the force terms onto the nearest grid cell
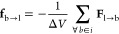
5
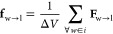
6Further details about the calculation/filtering of continuous phase volume fraction and the force terms can be found in the “Interphase coupling” section of our previous work.^23^

The motion of bubbles, when treated as discrete objects, can be tracked using Newton’s second law
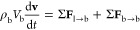
7ρ_b_ is kept constant as the flow is incompressible. According to the equation, the bubble of volume *V*_b_ moves at a velocity of **v**. The bubble motion is influenced by two forces: Σ**F**_l→b_ being the total force exerted by the liquid phase and Σ**F**_b→b_ being the collision force with other bubbles. Σ**F**_l→b_ can be decomposed into drag, lift, pressure, gravity, and the virtual mass force terms. The definition of these terms can be found in Table 1.

**Table 1:** Various Force Models^23,30,31^

forces (**F**_l → b_)	closures
**F**_G_ = ρ_b_*V*_b_**g**	
**F**_P_ = −V_b_∇*p*	
	(30) 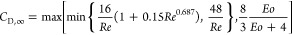
**F**_L_ = −C_L_ ρ_l_*V*_b_ (**v** – **u**) × ∇ × **u**	
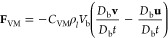	*C*_VM_ = 0.5

Courant number (*Co*) of the simulations is kept around the same value of 0.3 by changing the Lagrangian time step (Δ*t*_b_) in each step. A soft-sphere approach is applied to the bubble collision force (Σ**F**_b→b_). This approach employs a spring-dashpot model, in which the spring constant is treated as the surface tension of the bubble i.e., *k_n_* ∼ 2πσ.^27^ According to Xue et al.^27^ the spring constant and other mechanical properties should take the values as shown in Table 2. In general, collision models help improve the robustness of results by avoiding any unphysical overlapping of bubbles.^28,29^

**Table 2:** Collision Settings for Soft-Sphere Approach^27^

parameter	value
Young’s modulus *E*	50 N/m^2^
Poisson’s ratio ν	0.5
coefficient of restitution *e*	0.95
friction coefficient μ	0.09

The volume of fluid (VOF) method is used to capture the free surface at the top of a bubble column, through which bubbles leave the liquid. The definition of continuous phase volume fraction (α_c_) is expanded upon, to couple VOF with CFD-DPM. The continuous phase fraction (α_c_) comprises the volume fraction of the liquid phase (α_l_) and that of the gaseous phase (α_g_) above the free surface. Lagrangian bubbles (α_d_) transform to the continuous gas phase (α_g_) near the free surface. Definitions of α_c_ and α_l_ are given as,
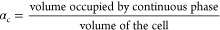
8
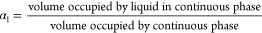
9The following phase fractions must add to unity
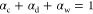
10
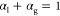
11The resultant interface capturing equation (VOF) for the continuous liquid phase is
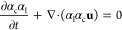
12Continuous phase density and viscosity in eqs 1 and 2 are given as
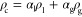

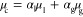
Hydrodynamic performance and mesh dependency of this numerical framework have been justified in our previous work.^23^

### Wire–Bubble Interactions

Wang et al.^15^ had remarked that the bubbles maintain a certain distance from the wires via a thin liquid film. Thus, the bubbles sense the wires indirectly. In order to include the effects of the wire mesh, we treat them as stationary solid Lagrangian particles. The presence of the wire mesh is included in eqs 1 and 2 by introducing two terms, α_w_ the volume fraction of the wire mesh, and (**f**_w→l_) additional forcing term due to wire mesh. The calculation of α_w_ is similar to that of α_d_. While the forcing term (**f**_w→l_) is calculated by filtering the summation of the forces exerted by the individual wires
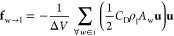
13where *A*_w_ is the projected area of the wire in the direction of liquid flow. The drag coefficient takes the following form
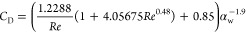
14which was obtained by Segers et al.^32^ using direct numerical simulation data of flow around crossing cylinders.

### Coalescence and Breakup

Bubble coalescence and breakup are two important phenomena that decide the bubble size distribution in a column. Sommerfeld et al.^33^ proposed a model accounting for coalescence. According to their work, bubbles coalesce when the bubble collision time is longer than its counterpart, film drainage time. The film drainage time *t*_drain_ is determined by the Prince and Blanch^34^ model
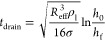
15where *h*_0_ and *h*_f_ are the initial and final film thicknesses, respectively. In this work, *h*_0_/*h*_f_ is taken as 10^–4^ as used by Sommerfeld et al.^33^ The effective diameter, *R*_eff_, is the harmonic mean of the radii of the two interacting bubbles. σ is the surface tension coefficient, and ρ_l_ is the liquid density.

Lau^35^ proposed a model for addressing bubble breakup. Breakup occurs when bubble deformation due to inertial forces exceeds the restoring force due to surface tension. This competition of two forces can be rewritten as an inequality between the Weber number and its critical value. It is expressed as follows
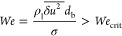
16where  is the mean square velocity difference over the bubble diameter *d*_b_. *We*_crit_ = 12 is the most frequently used value for breakup due to hydrodynamic stability. The bubble breaks into two daughter bubbles as per a U-distribution as proposed by Luo & Svendsen^36^ and Tsouris & Tavlarides.^37^ This means that the probability curve of the relative daughter bubble size is U-shaped, yielding mostly one large and one small bubble.

### Species and Interphase Mass Transfer

One of the case studies involves the chemisorption of CO_2_ in an aqueous NaOH solution. The whole process covers the physical absorption of CO_2_ in the aqueous phase, followed by two consecutive reversible reactions as detailed in Darmana et al.^38^

The species transport equation of a chemical species *j* with a mass fraction of *Y*_l_^j^ in the fluid is written as
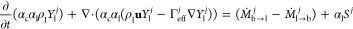
17where *S^j^* is the source term accounting for the production or consumption of species *j* due to homogeneous chemical reactions as modeled in Darmana et al.^38^ Γ_eff_^*j*^ is the effective transport coefficient defined by
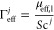
where *Sc*^*j*^ is the Schmidt number, , and *D*_*j*_ is the diffusivity of species *j*.

*Ṁ*^*j*^ represents mass transfer at the bubble–liquid interface. The interphase mass transfer is considered to be driven by the mass fraction gradient over the bubble–liquid interface. The mass fractions of transferred species *j* in the liquid bulk and bubble are represented by *Y*_l_^*j*^ and *Y*_b_^*j*^, respectively, whereas *Y*_l_^j*^ and *Y*_b_^j*^ are the mass fractions at the liquid and bubble sides of the bubble–liquid interface, respectively. The interphase mass transfer *Ṁ*^*j*^ from a bubble with an interfacial surface area of *A*_b_ is thus given as
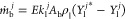
18where *E* is the mass transfer enhancement factor due to chemical reactions, which is estimated using the same correlation as in Jain et al.^29^ for CO_2_ chemisorption in NaOH solution. *k*_l_^*j*^ is the mass transfer coefficient for species *j*. The mass fraction on the liquid side of the interface can be determined using Henry’s law
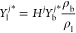
where *H*^*j*^ is the Henry constant for species *j*.

### Bubble Cutting Model

In our previous work,^24^ a closure model has been established for accurate prediction of the outcome (cutting/passage/stuck) of bubble–wire mesh interaction. This model is based on the energy balance of a bubble. We evaluate the excess dimensionless energy Δ*E* available for cutting/passage by comparing the change in kinetic energy plus the work due to drag and virtual mass (first term in RHS) and the dimensionless surface energy (*Eo*_*t*_).
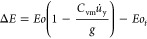
19The contribution of virtual mass is approximated as
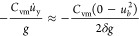
20by assuming a constant deceleration *u̇*_y_ over a distance, δ, which we term the deceleration thickness. δ = 0.25*d*_b_ was roughly determined for the cases with a liquid viscosity of 80 mPas as a preliminary observation from the DNS work of Baltussen.^14^ In the following paragraph, we present a physics-based estimate of this thickness.

**Figure 1:**
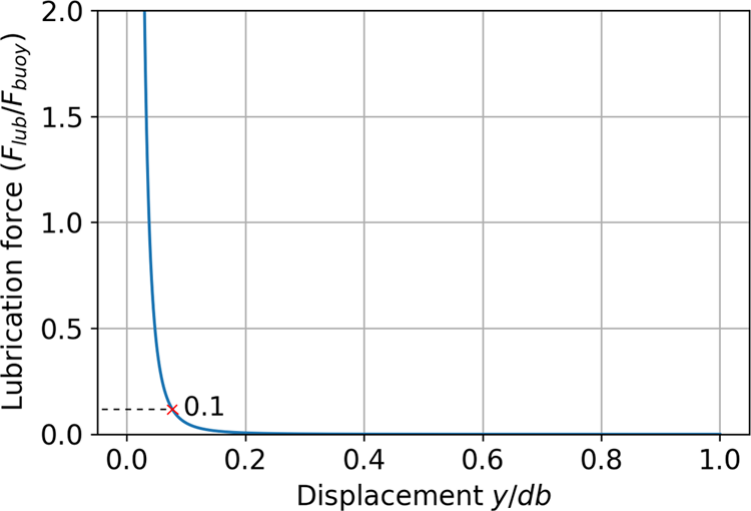
Lubrication force for bubble of diameter 4 mm, a grid opening size of 3.7 mm, *d*_w_ = 0.55 mm, and a kinematic viscosity of 1e-6 m^2^/s.

Zhang et al.^39^ illustrate the thin-film dynamics of a bubble impinging on a solid wall. When a rising bubble is very close to the wall, the net vertical force due to the pressure distribution on the bubble area facing the solid surface is given by
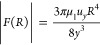
21where *y* is the film thickness between the bubble and the wall, μ_l_ is the liquid viscosity, *u_y_* is the bubble velocity, and *R* is the bubble radius. In our case, the bubble does not hit a solid wall but a wire mesh with openings. To account for the openings, we correct using the relative projected area of the wires, ε_w_.
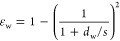
22The net force on the bubble is expressed as
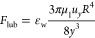
23Figure 1 shows an example of the lubrication force (normalized by the buoyancy force *F*_buoy_) as a function of the bubble–wire distance (normalized by the bubble diameter). As seen in the plot, the bubble feels the effect of the wires around *F*_lub_/*F*_buoy_ = 0.1 as the curve grows steeply after that. As the drop of normalized force is highly dependent on the distance (1/*y*^3^), the other parameters in eq 23 can be ignored while calculating the point of contact. The corresponding δ is found by back-substituting this value of *F*_lub_/*F*_buoy_ = 0.1 to eq 23
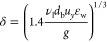
24

It is clear that this thickness is not a constant but dependent on the liquid properties, wire thickness, grid opening, and the bubble terminal velocity. In the upcoming sections, we use eq 24 to calculate the virtual mass term.

## MSBC Simulations

With the refined derivation as explained in the previous section, we implemented the cutting model into the CFD-DPM-VOF framework for MSBC simulations. The main objective is to evaluate the cutting model in its ability to predict the bubble–wire interaction outcome. To this end, experiments recently reported by Chen et al.^12^ and CO_2_ chemisorption experiments done by Sujatha^13^ are chosen as the case studies. The first case uses a rectangular BCR with dimensions of 120 mm × 25 mm × 840 mm (W × D × H). A single wire mesh was fixed 150 mm above the gas inlet, and different experiments were performed for liquid viscosity varying from 1 to 39.6 mPa·s and using two mesh openings (3.8 and 5.5 mm). Experiments in the second case were conducted in a pseudo-two-dimensional (2D) BCR of dimensions 200 mm × 30 mm × 1300 mm (W × D × H) with a single wire mesh fixed at a distance of 260 mm from the bottom of the column. The simulation parameters of the two cases are given in Table 3.

**Table 3:** Simulation Parameters

parameter	case 1	case 2
liquid density, kg/m^3^	1000	1000
gas density, kg/m^3^	1	1
shear viscosity, mPa·s	1–40	1
surface tension, N/m	0.073	0.073
initial liquid pH	7	12.5
gas superficial velocity, mm/s	3.5	15
initial bubble diameter, mm	4.5–5.0	4.5
wire diameter, mm	0.55	0.55
mesh opening, mm	3.8, 5.5	3.7
domain, m^3^	0.12 × 0.025 × 0.84	0.14 × 0.03 × 0.72
initial liquid height, m	0.6	0.6
wire mesh height, m	0.15	0.26
number of nozzles	15 × 1	15 × 1
pitch of nozzles, m	0.008	0.01

### Case Chen et al.

**Figure 2:**
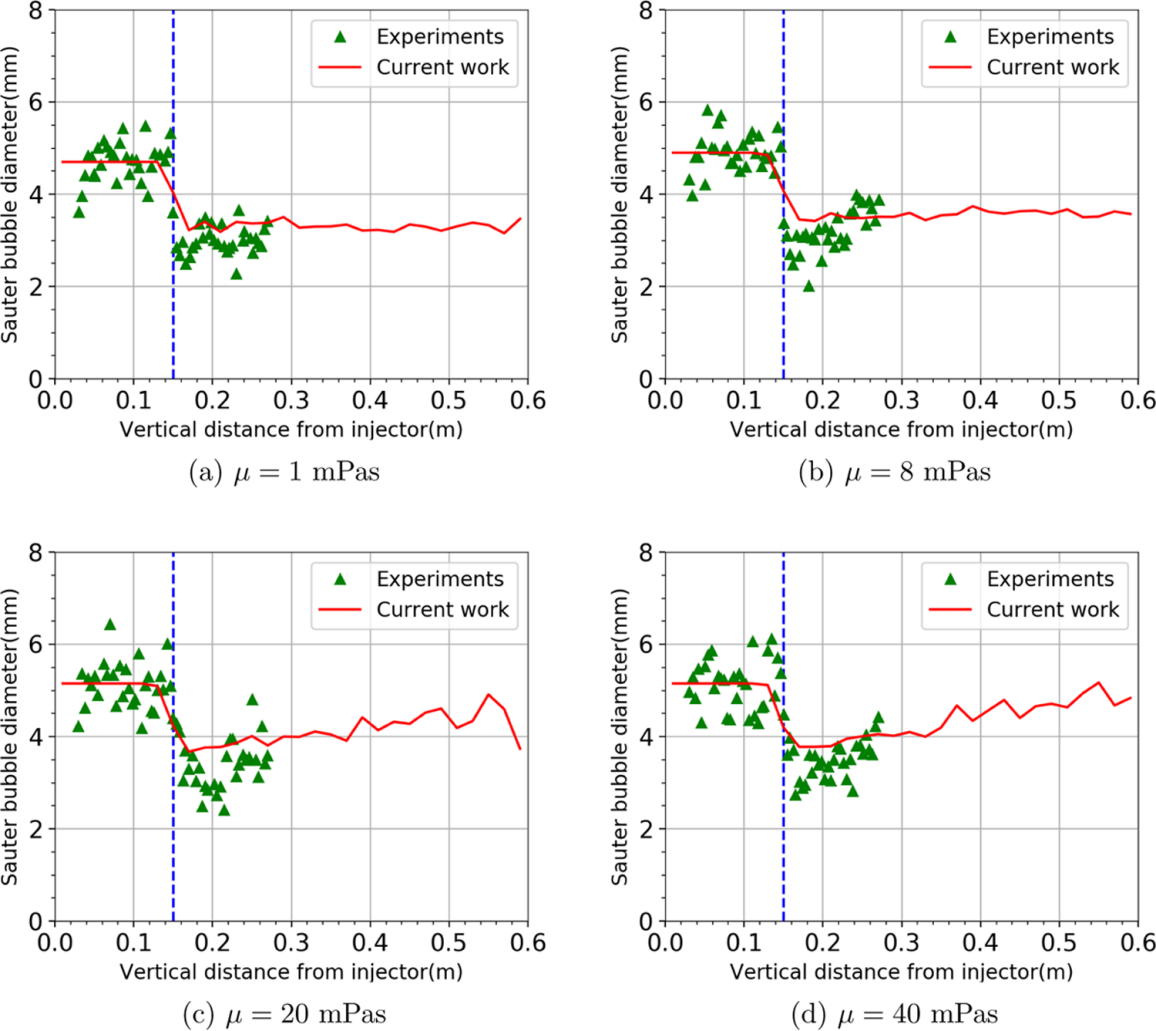
Sauter mean diameter *d*_32_ as a function of column height with an *s* = 3.8 mm mesh opening at a superficial gas velocity of 3.5 mm/s. Simulation results (solid line) are compared to the experimental data (symbols). The dashed line indicates the location where the wire mesh is fixed.

In this section, we present the simulation results of the MSBC studied experimentally by Chen et al.,^12^ which also investigated the effect of liquid viscosity on bubble cutting. Figure 2 shows a comparison of Sauter mean diameter *d*_32_ between the simulation results and experimental data with a *s* = 3.8 mm mesh opening for different liquid viscosities. Overall, we can see that the predictions of *d*_32_ from the simulations agree very well with the experimental measurement. Not only is the effect of the bubble cutting well modeled but also is the impact of viscosity. There is a significant decrease in the Sauter mean diameter across the wire mesh. Bubbles are successfully cut by the mesh into smaller bubbles. As for the effect of viscosity, the *d*_32_ increases with the liquid viscosity both before and after bubble cutting. This is expected as shown in eq 24, as the viscosity increases the deceleration thickness δ, thus lowering the virtual mass energy during interaction with the wire mesh. For lower viscosity (μ = 1 and 8 mPa.s), *d*_32_ stabilizes after cutting at a value of around 3 and 3.5 mm, respectively. Similar to the observations by Chen et al.,^12^ the bubble size distribution is independent of the vertical height of the column. This is most likely due to the dynamic equilibrium state achieved from bubble coalescence and the breakup in the bubbly flow. On the contrary, for high viscosity (μ = 20 and 40 mPa.s), *d*_32_ continues to increase after cutting along the column height due to a more pronounced coalescence (longer residence time of bubbles in the column).

**Figure 3:**
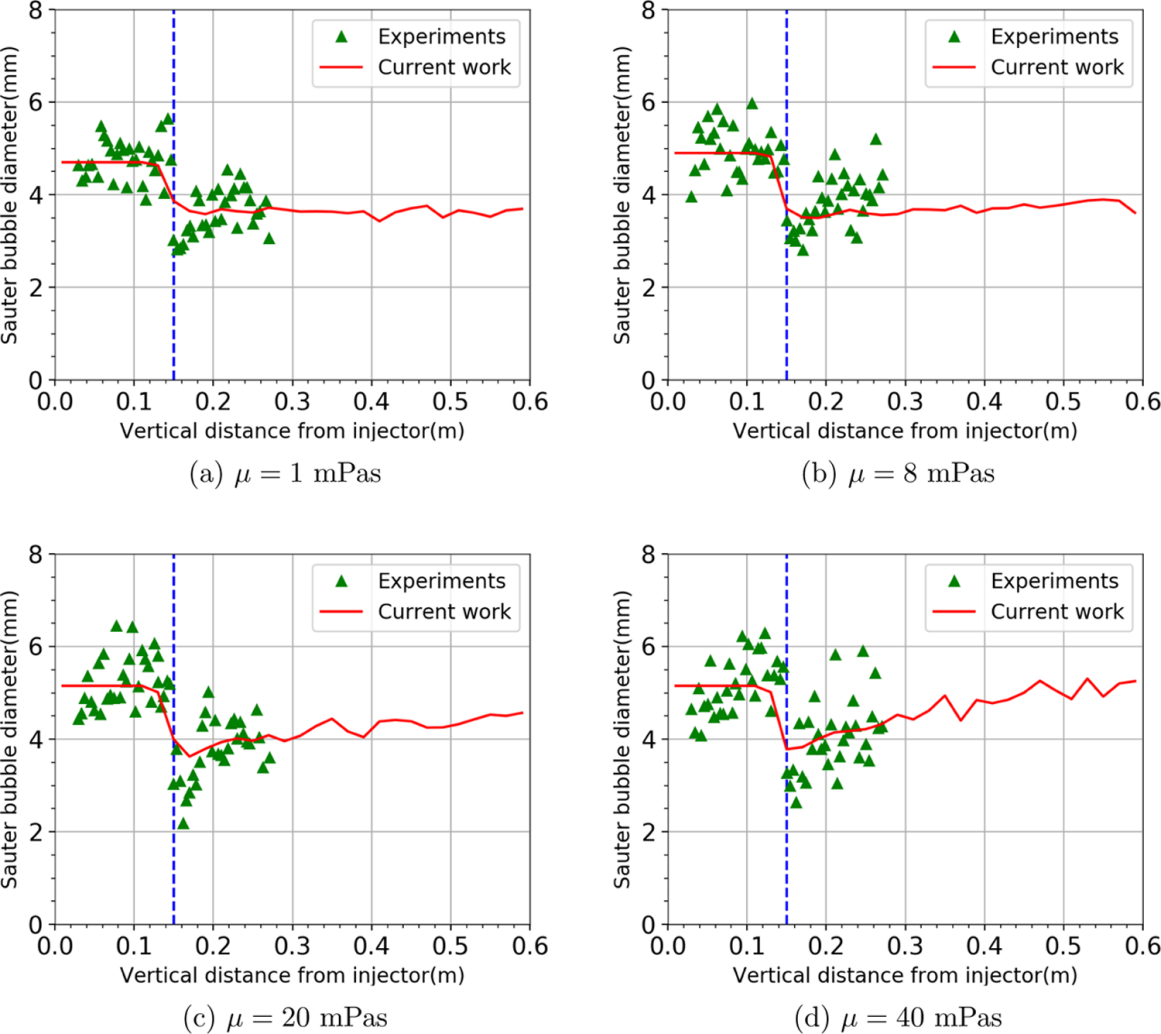
Sauter mean diameter *d*_32_ as a function of column height with an *s* = 5.5 mm mesh opening at a superficial gas velocity of 3.5 mm/s. Simulation results (solid line) are compared to the experimental data (symbols). The dashed line indicates the location where the wire mesh is fixed.

**Figure 4:**
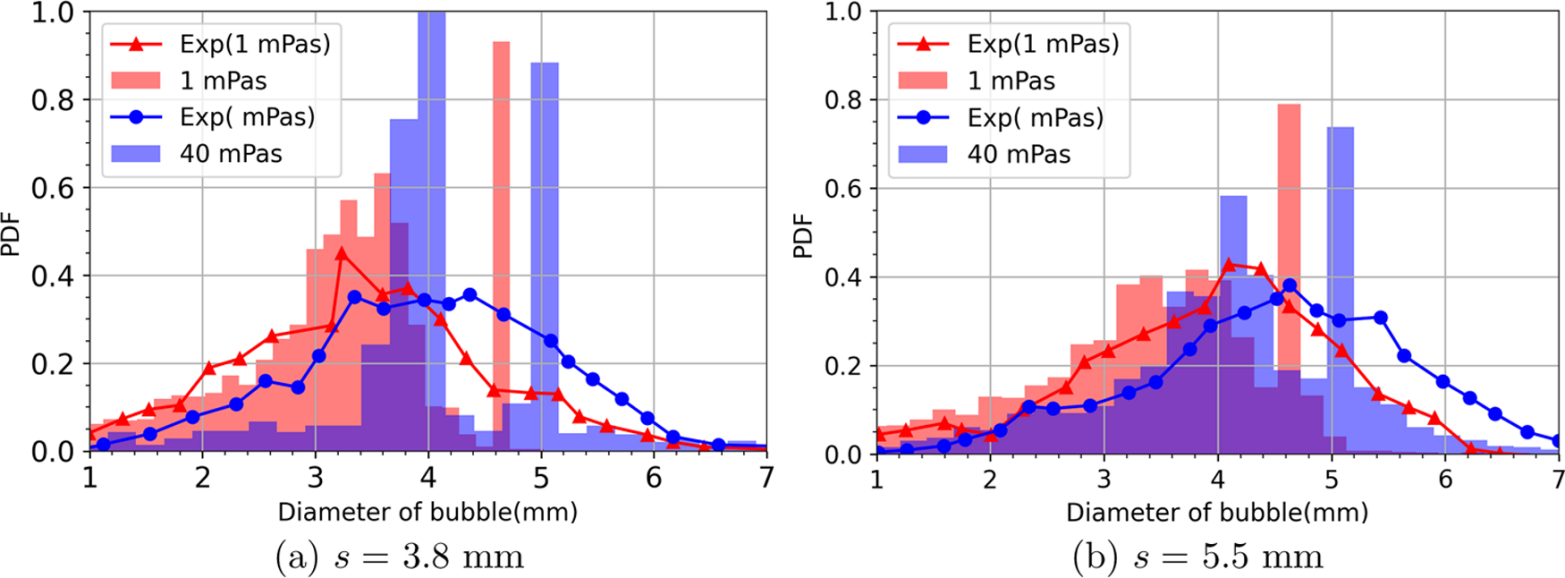
Probability density function (PDF) of bubble size distribution for the cases with two grid openings (a, b) and viscosity.

Figure 3 shows the same comparison, but for the larger mesh opening, *s* = 5.5 mm. Similar trends as in Figure 2 can be observed. The cutting model accurately describes the bubble cutting well, giving the correct apparent change in bubble Sauter mean diameter. In the case of the 3.8 and 5.5 mm mesh openings, the Sauter mean diameter after cutting is approx. 30.8 and 24.7% smaller than before cutting, respectively. Figure 4 further shows the bubble size distribution for two different liquid viscosities for the two mesh openings. Experimental data indicates that for both mesh openings, the bubble size distribution gets slightly wider and the main peak in bubble size shifts slightly to the right as the liquid viscosity increases from 1 to 40 mPas. A similar effect of the liquid viscosity on the bubble size distribution can be seen from our simulation results. However, in our case, the bubbles are injected at either 4.5 or 5.5 mm, thus having a spike in these areas. On the other hand, this is not the case in the experiments as the initial bubble size has a certain (unknown) distribution near the injector. As our main focus with the simulation is to observe cutting behavior, we do not address this any further.

### Case Sujatha

**Figure 5:**
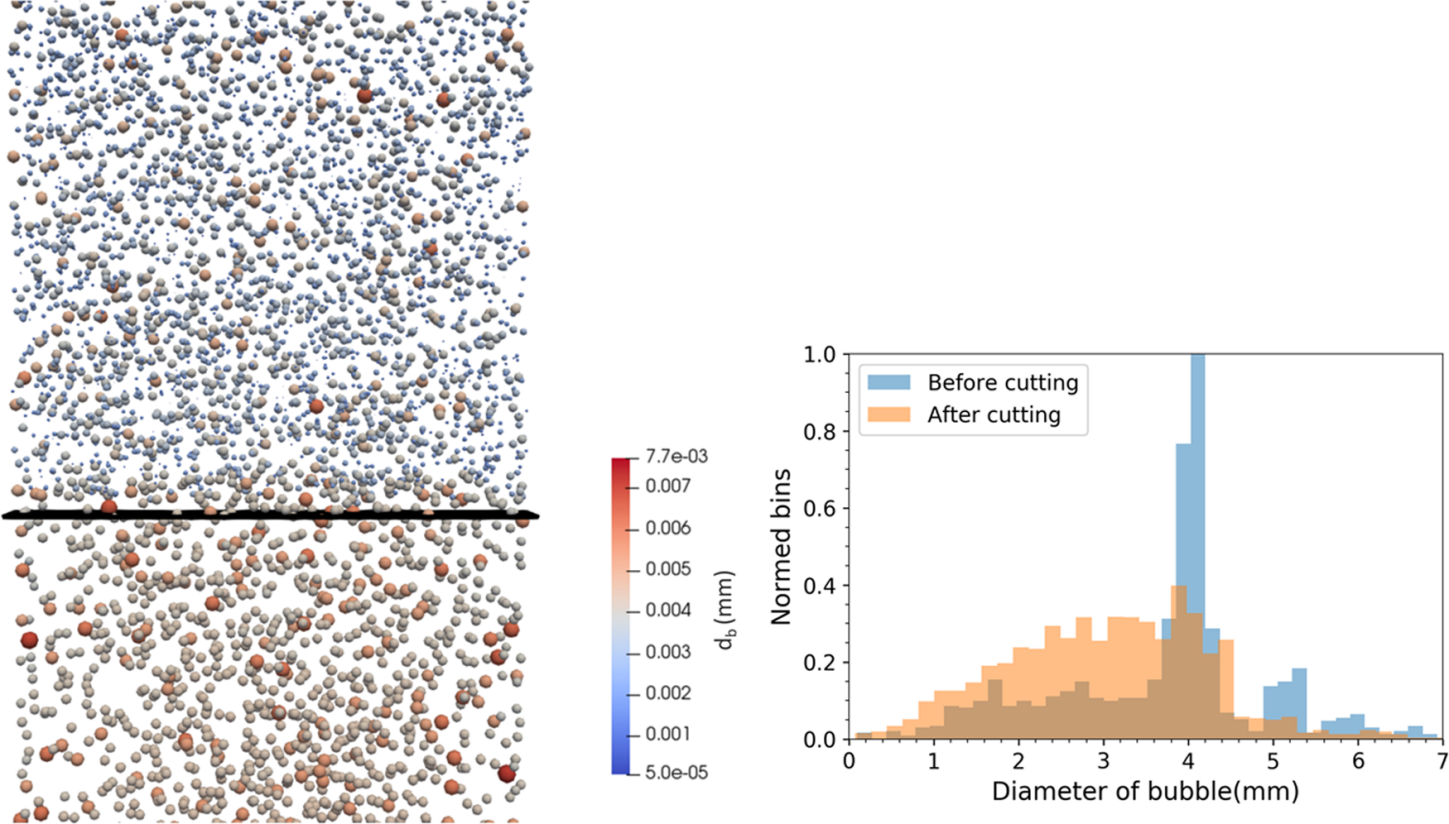
Snapshot of bubble cutting simulations of Sujatha^13^ case (left) and instantaneous bubble size distribution before and after cutting (right) at *t* = 2.6 s.

**Figure 6:**
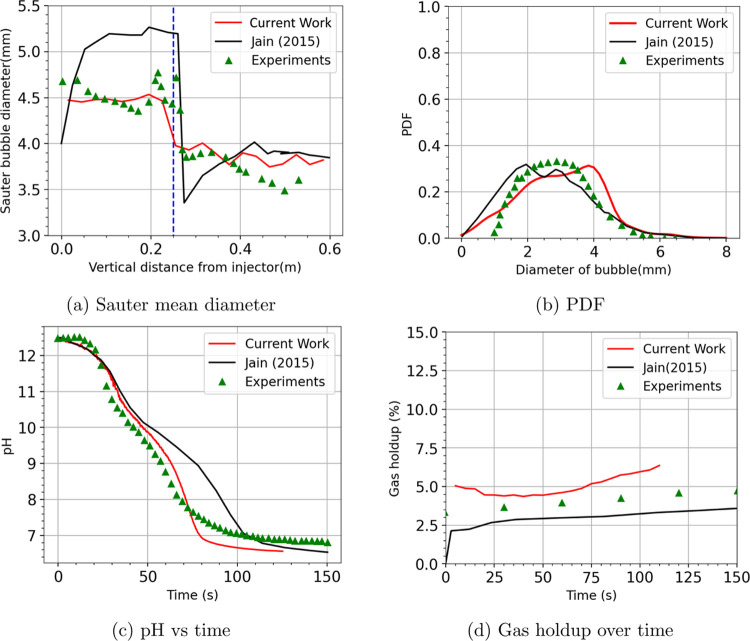
Comparison between the current simulation, simulation done by Jain et al.,^29^ and experiments done by Sujatha^13^ for (a) the Sauter mean diameter, (b) probability density function (PDF) of bubble size in the region of 0.42 < *z* < 0.6 m, (c) pH evolution, and (d) the overall gas holdup.

In this section, we present the simulation results of CO_2_ chemisorption in an MSBC and compare them with the experimental data of Sujatha^13^ and the numerical data of Jain et al.^29^Figure 6 compares the current simulation results with the experimental data and the previous simulation results using a simple geometrical cutting model.^29^Figure 6a displays the Sauter mean diameter averaged in the time interval 10–15 s as a function of height from the bubble injector. First of all, it is clear that our current simulation gives a significantly improved prediction of the bubble diameter both before and after cutting, compared to the model of Jain et al.^29^ Looking closely at the current simulation results and the experimental data, we see a very good match of the diameters immediately before and after the cutting, i.e., 4.5 to 3.9 mm. Together with the demonstration from the other case in Section 3.1, this gives us confidence in the current cutting model. Figure 5 shows a snapshot of bubbles and the instantaneous bubble size distributions adjacent to the wire mesh (before and after cutting). The functioning of our cutting model is clearly visualized in the two figures. Just before cutting, the bubble size peaks between 4 and 5 mm. After cutting, the size distribution is broader, and the mean bubble diameter shifts to around 3 mm.

In Figure 6a, slight deviations between our simulation and experimental results are present in the injection area and near the free surface. In the injection area, the bubble size in our simulation appears to stay almost constant with height. This indicates an equilibrium state due to balancing mass transfer (CO_2_ absorption), breakup, and coalescence. Experimental measurement shows a slight decrease of the Sauter diameter in this area; however, there also exist quite some fluctuations. After cutting, the Sauter diameter keeps decreasing until the free surface in experiments, implying that CO_2_ absorption is more pronounced than coalescence. This reduction is, however, marginal in our simulation results suggesting an underestimation of the chemisorption. Figure 6b displays the probability density function of the bubble diameter in this region at a column height of 0.42–0.6 m. The simulation results in a larger size distribution than the experiments. Note that the simulation results of Jain et al.^29^ present a similar trend in the injection and free surface regions. All of these deviations might imply that the bubble coalescence and breakup models which are the same as in the simulations of Jain et al.^29^ need to be improved.

Figure 6c,6d displays the pH value and gas holdup over time. Compared to the simulation of Jain et al.,^29^ our current results again have a better agreement to the experimental data, as a result of a better-predicted bubble size after cutting. The present simulation can capture the two inflection points in the drop of [OH]^−^ ions. The slight overprediction of gas holdup may be due to the contribution of the coalescence and breakup model as discussed above.

## Design of Wire Meshes

**Figure 7:**
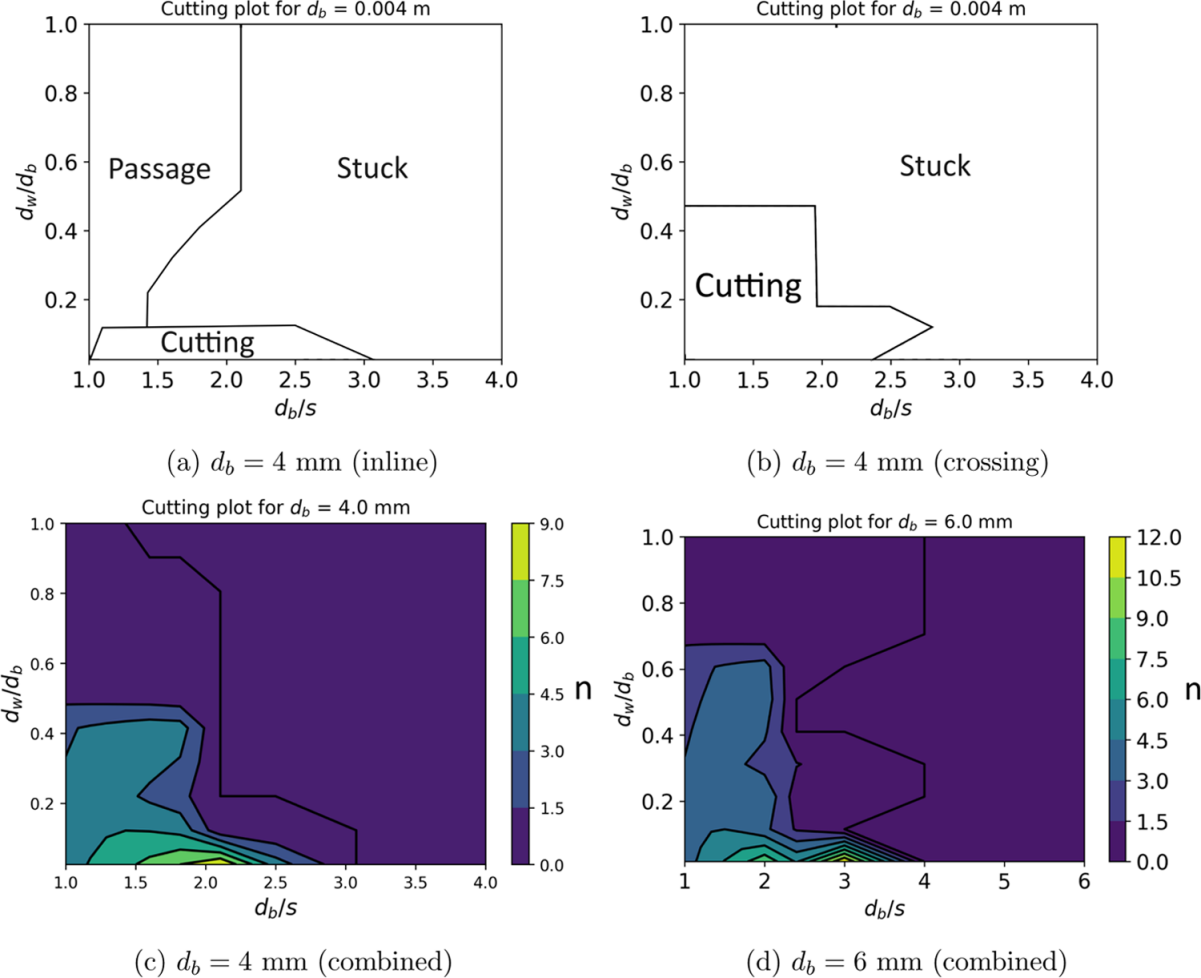
Regime diagrams for bubble impacting meshes with different grid openings and grid thicknesses, *d*_b_/*s* and *d*_w_/*s*. Color map of the number of daughter bubbles combined for the three configurations.

In previous sections, we have validated our bubble cutting model with two experimental case studies involving both high and low superficial gas velocities as well as low to high liquid viscosity. We now demonstrate how to use this cutting model for the preliminary design of wire meshes. The design ought to optimize the bubble-mesh interaction in order to achieve a reduced average bubble size so that the mass transfer is enhanced. This can be achieved by assuming a constant initial bubble diameter (*d*_b_) and varying the grid opening (*s*) and wire thickness (*d*_w_) to calculate the number of daughter bubbles produced (*n*). In order to calculate the value of *n*, we computed the threshold energies (*Eo*_*t*_) for different bubble-mesh configurations and different values of *d*_b_/*s* and *d*_w_/*d*_b_ using an energy balance as outlined by Subburaj et al.^24^ If the excess energy of the bubble is positive (Δ*E* > 0), then we have a cut condition. Otherwise, the bubble is stuck (*n* = 0). We apply these conditions for all of the three different configurations: bubbles approaching a single wire (i), a mesh opening (o), or two crossing wires (c). Then, we combine them with a probability model^24^ (*P*_*i*_, *P*_*o*_, *P*_*c*_) to produce the expected *n̂*. The obtained results are plotted in Figure 7. We observe that increasing *d*_b_/*s* alone increases the number of daughter bubbles until a certain point after which the bubble gets stuck. When we increase the wire thickness, it only introduces more resistance to cutting. So a general trend to maximize cutting is to have a lower *d*_w_/*d*_b_ and a moderate *d*_b_/*s*. For the case of a bubble with *d*_b_ = 4 mm, the optimal *d*_b_/*s* is 1.5–2.5, where we can produce 9 bubbles on average. Although our study concludes that the *d*_w_/*d*_b_ has to be small, having a moderate thickness helps avoid premature coalescence right after cutting thus maintaining a stable bubble distribution. We do not explore this aspect in this work.

## Concluding Remarks

In this work, we test the performance of our previously developed bubble cutting model in Euler–Lagrange simulations of microstructured bubble columns. This cutting model was established based on an energy analysis of a bubble interacting with a wire mesh.^24^ We further refine the cutting model by introducing a deceleration thickness δ, which is dependent on liquid properties (kinematic viscosity) and the wire thickness according to the lubrication theory. Subsequently, this cutting model is implemented in the CFD-DPM framework with an OpenFOAM-based solver. Two experimental works by Chen et al.^12^ and Sujatha^13^ are employed as validation cases, which consider both high and low gas superficial velocity, as well as different liquid viscosities.

Overall, with the current cutting model, our simulations quantitatively reproduce these experimental measurements of bubbly flow, including the bubble size (distribution) and gas holdup. The reduction in cutting due to an increase in viscosity is captured well due to an accurate deceleration thickness. This suggests that the initial impact of the bubble plays an important role in cutting. In particular, for the case of Sujatha,^13^ our model shows better performance than the geometric cutting model proposed by Jain et al.^29^ As a consequence of a better bubble dynamics modeling, the simulation also gives a better agreement to the experiments on the chemical conversion performance. Bubble coalescence and breakup play an important role in cases with higher superficial gas velocity, for which there is still room for improvement in modeling.

Last, we address a few points on the limits/improvement of the current cutting model for future development:

- the energy analysis is based on three primary impact configurations (inline, single wire, crossing), whereas in practice a bubble can come into contact with a wire mesh in an arbitrary configuration;
- when cutting a bubble, the number of daughter bubbles is estimated by assuming a square projection area. However, in reality, the projected area is better approximated as spherical.
- it is observed that in the simulations bubble cutting sometimes leads to very small bubbles (*d*_*b*_ < 0.5 *mm*). Such small bubbles pose a challenge to the soft-sphere collision model as it demands a very small time step (*O*(Δ*t*) < 10^–7^s). Treating collisions of small bubbles using a hard-sphere model may be a way to solve this issue.
